# Reappraising the microscopic anatomy of human testis: identification of telocyte networks in the peritubular and intertubular stromal space

**DOI:** 10.1038/s41598-018-33126-2

**Published:** 2018-10-03

**Authors:** Mirca Marini, Irene Rosa, Daniele Guasti, Mauro Gacci, Eleonora Sgambati, Lidia Ibba-Manneschi, Mirko Manetti

**Affiliations:** 1 Department of Experimental and Clinical Medicine, Section of Anatomy and Histology, University of Florence, Florence, Italy; 2 Department of Minimally Invasive and Robotic Urologic Surgery and Kidney Transplantation, University of Florence, Florence, Italy; 3 Department of Biosciences and Territory, University of Molise, Pesche, Isernia, Italy

## Abstract

Telocytes are a recently described stromal cell type widely distributed in various organs including the female and male reproductive systems. This study was aimed to investigate for the first time the existence, distribution and characteristics of telocytes in normal human testis by an integrated morphological approach (immunohistochemistry, immunofluorescence and transmission electron microscopy). We found that telocytes displaying typical long and moniliform prolongations and coexpressing CD34 and PDGFRα formed networks in the outer layer of peritubular tissue and around Leydig cells and vessels in the intertubular stroma. Testicular telocytes were immunophenotypically negative for CD31, c-kit/CD117 as well as α-SMA, thus making them clearly distinguishable from myoid cells/myofibroblasts located in the inner layer of peritubular tissue. Transmission electron microscopy confirmed the presence of cells ultrastructurally identifiable as telocytes (*i.e*. cells with telopodes alternating podomers and podoms) in the aforementioned locations. Intercellular contacts between neighboring telocytes and telopodes were observed throughout the testicular stromal compartment. Telopodes intimately surrounded and often established close contacts with peritubular myoid cells/myofibroblasts, Leydig cells and vessels. Extracellular vesicles were also frequently detected near telopodes. In summary, we demonstrated that telocytes are a previously neglected stromal component of human testis with potential implications in tissue homeostasis deserving further investigation.

## Introduction

The adult human testes are components of both the reproductive and endocrine systems structurally designed to perform spermatogenesis (*i.e*. the production of haploid male gametes/spermatozoa from diploid postnatal germ-line stem cells) and elaborate male sex hormones (androgens), primarily testosterone^1^. Microscopically, the testis parenchyma is organized into multiple conical lobules each consisting of up to four extremely convoluted seminiferous tubules surrounded by the intertubular stromal (interstitial) compartment, a highly vascularized loose connective tissue containing groups of endocrine Leydig cells and additional cellular elements such as macrophages and fibroblasts^1^. Each seminiferous tubule is composed of an inner layer of germinal epithelium featuring male germ cells in different developmental stages harbored within invaginations of supporting Sertoli cells, and an external layer of peritubular tissue (also referred to as lamina propria) separated by a basement membrane^1,2^. Such peritubular connective tissue appears populated by the so-called myoid cells, a kind of myofibroblasts which are supposed to be responsible of the peristaltic contractions of the seminiferous tubules essential for the transport of the immotile spermatozoa to the rete testis located within the testicular mediastinum^1,3^.

During the last decades the interest of most testis research was focused on germ cells, Sertoli cells and testosterone-releasing Leydig cells, while the precise organization of the peritubular and intertubular spaces has been often neglected^1,4,5^. That is not surprising, because during the removal of testicular tissue the seminiferous tubules are shifted against each other so that the loose intertubular stroma frequently disrupts^5^. As a matter of fact, aside from Leydig cells, there is little information concerning the possible existence of different interstitial cell types in the human testis stromal compartment.

In recent years, the classic description of the microscopic anatomical structure of a variety of organs in vertebrates, including humans, has been challenged by the identification of a previously unrecognized, though widespread, interstitial cell type named telocyte (TC)^6–19^. On the basis of their distinctive morphological features, TCs are commonly defined as interstitial cells with telopodes, namely very slender and long-distance spreading moniliform cellular prolongations characterized by an alternation of extremely thin segments (podomers) and small dilated portions (podoms) clearly discernible by electron microscopy^6,7,20^. Besides their peculiar ultrastructural characteristics, growing evidence further indicates that TCs are rather different from other types of stromal cells based on cell surface antigens and gene expression, proteomic and microRNA signatures^6,7,16^. Functionally, TCs are arranged to build a three-dimensional network within the tissue interstitium where they are capable to exchange signals with neighboring cells either by direct intercellular contacts or by the release of exosomes and other types of extracellular vesicles^6,7,16,21–28^. Hence, TCs are increasingly being regarded as key cellular elements implicated in tissue morphogenesis and homeostasis, intercellular signaling, immune surveillance and guidance of tissue-resident stem/progenitor cell self-renewal and differentiation, as well as a variety of pathologies^6,7,15,16,19,29–39^. Indeed, the possible suitability of these newly described stromal cells for tissue reparative and regenerative purposes is currently attracting much interest^40,41^.

Recent works have uncovered the presence of TCs in organs of the female and male reproductive systems from different species of vertebrates^16,29,42–49^. To date, however, only one report has described the existence of TCs in the testis of the Chinese soft-shelled turtle^50^, while there are no studies on TCs in the mammalian and human testicular stroma. Therefore, in this study we aimed to investigate for the first time the existence, distribution and characteristics of TCs in normal human testis by an integrated morphological approach featuring immunohistochemistry, immunofluorescence and transmission electron microscopy analyses.

## Results

**Figure 1:**
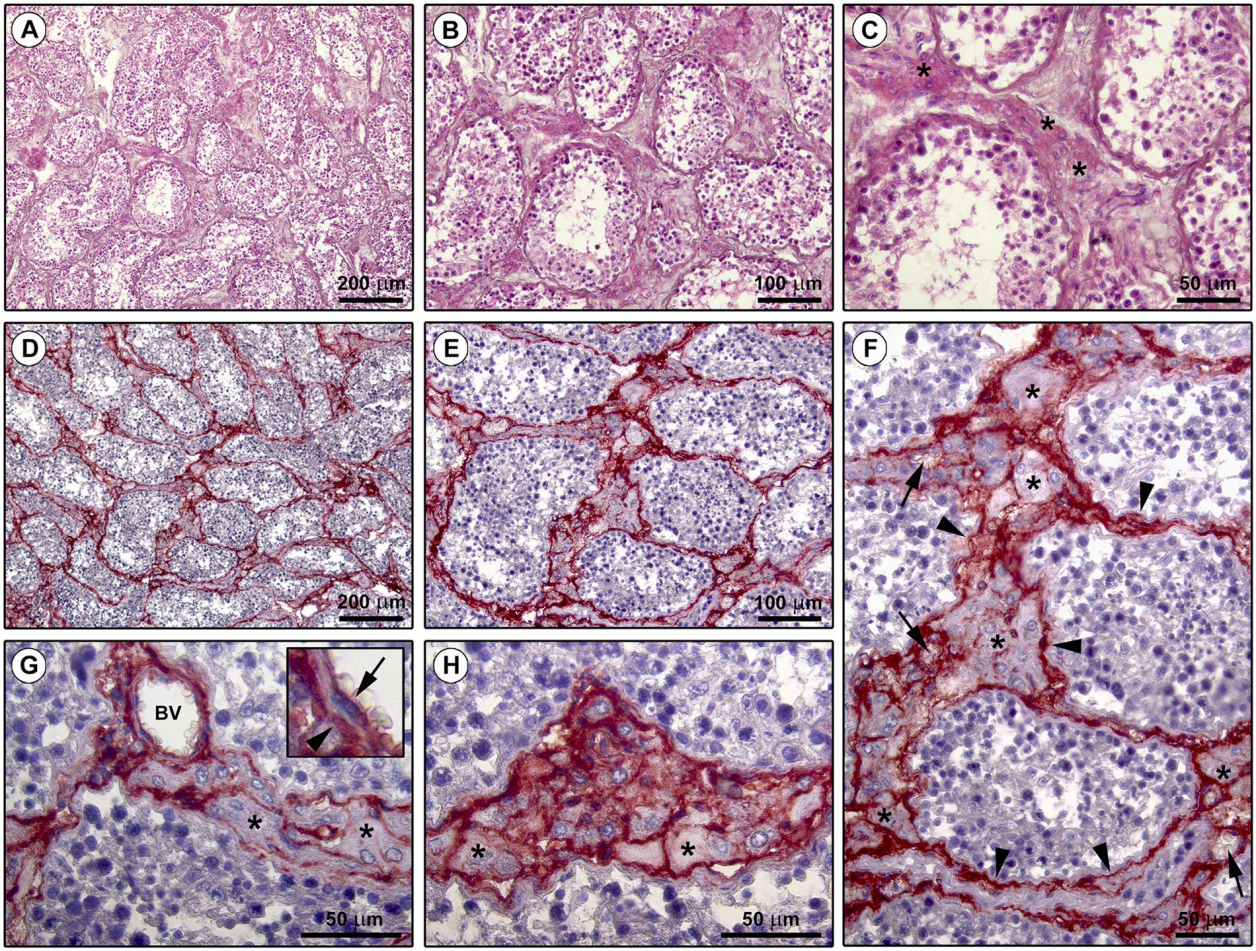
Light microscopy photomicrographs of human testis sections. (**A**–**C**) Representative photomicrographs of hematoxylin and eosin-stained tissue slides. Seminiferous tubules cut in various planes of section are surrounded by the peritubular and intertubular connective tissue (**A,B**). Note the optimal preservation of the peritubular and intertubular stroma, as testified by the presence of typical clusters of testosterone-secreting Leydig cells displaying an extensive eosinophilic cytoplasm (asterisks) in close vicinity to blood microvessels and embedded in an extracellular matrix (**C**). (**D**–**H**) Representative photomicrographs of testicular sections subjected to CD34 immunoperoxidase-based immunohistochemistry with hematoxylin counterstain. CD34-positive interstitial cells (red-stained) form a continuous network throughout the peritubular and intertubular stromal spaces (**D,E**). At higher magnification, the CD34-positive interstitial cells exhibit an elongated/spindle morphology with long and thin varicose cytoplasmic prolongations surrounding the seminiferous tubules (arrowheads in **F**), as well as the Leydig cells (asterisks in **F**–**H**) and blood microvessels (arrows in **F**) in the intertubular stroma. CD34 immunoreactivity is observed also in endothelial cells of blood vessels (BV); a higher magnification of CD34-positive endothelial cells lining the vessel lumen (arrow) and CD34-positive interstitial cells bordering the abluminal side of the vessel basement membrane (arrowhead) is shown in the inset (**G**). The CD34-positive interstitial cells constitute a complex reticular network around the Leydig cells (asterisks) in the intertubular stromal tissue (**H**). Scale bar: 200 µm (**A,D**), 100 µm (**B,E**), 50 µm (**C,F–H**).

**Figure 2:**
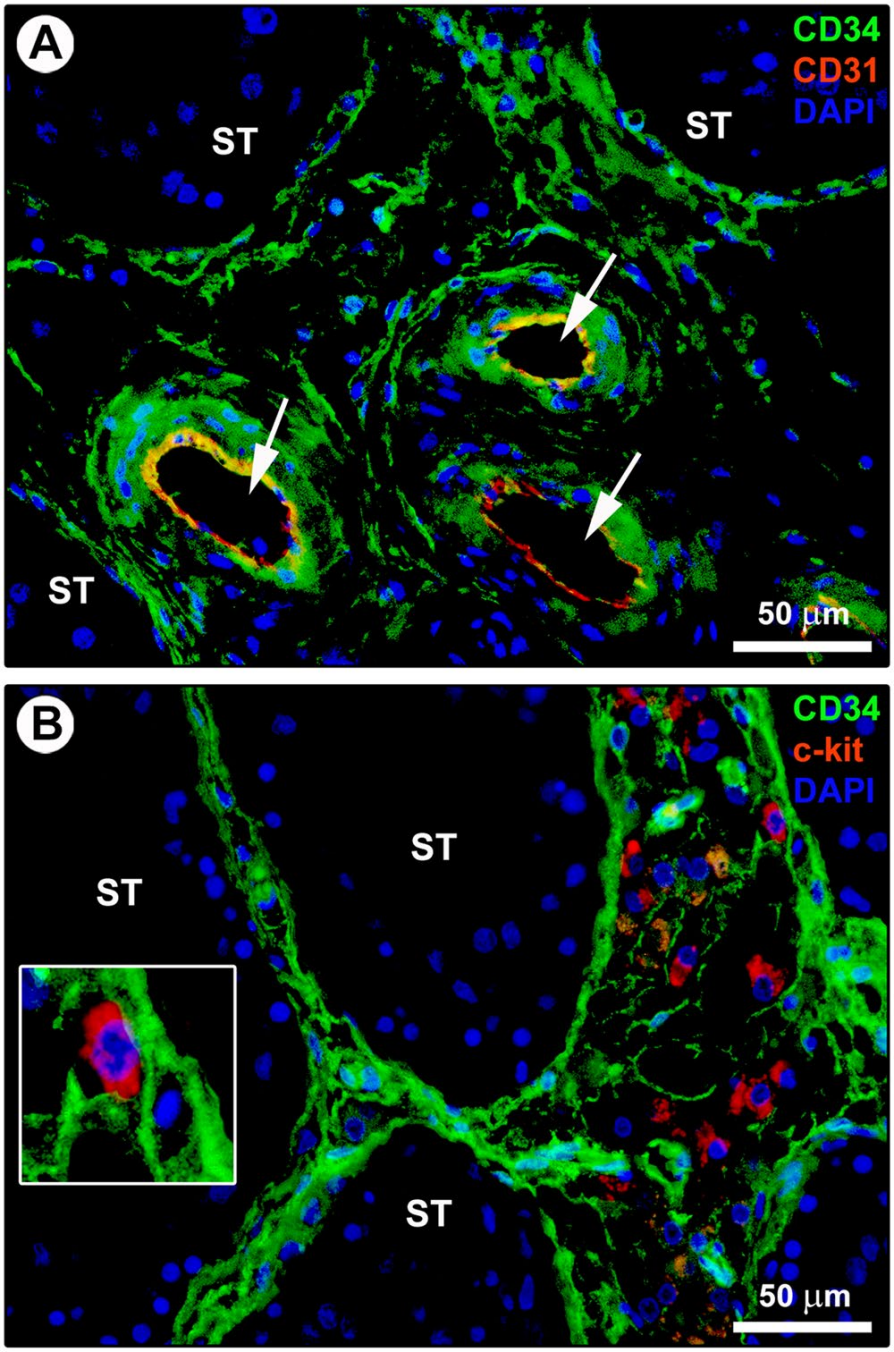
Double immunostaining of human testis sections. (**A**) Representative photomicrograph of immunofluorescence labeling for CD34 (green) and CD31 (red) with 4′,6-diamidino-2-phenylindole (DAPI; blue) counterstain for nuclei. The CD34-positive interstitial cells surrounding the seminiferous tubules (ST) and forming a reticular network in the intertubular stromal tissue lack CD31 immunoreactivity. The endothelial cells lining the lumen of microvessels (arrows) are CD34-positive/CD31-positive. (**B**) Representative photomicrograph of immunofluorescence labeling for CD34 (green) and c-kit/CD117 (red) with DAPI (blue) counterstain for nuclei. The CD34-positive interstitial cells distributed in the peritubular and intertubular stroma are immunophenotypically negative for c-kit/CD117. Immunoreactivity for c-kit/CD117 is detected in oval/round-shaped cells, presumably Leydig cells and/or mast cells, surrounded by the network of CD34-positive interstitial cells. A higher magnification of a c-kit/CD117-positive cell embraced by the cytoplasmic processes of CD34-positive interstitial cells is depicted in the inset. Scale bar: 50 µm (**A,B**).

**Figure 3:**
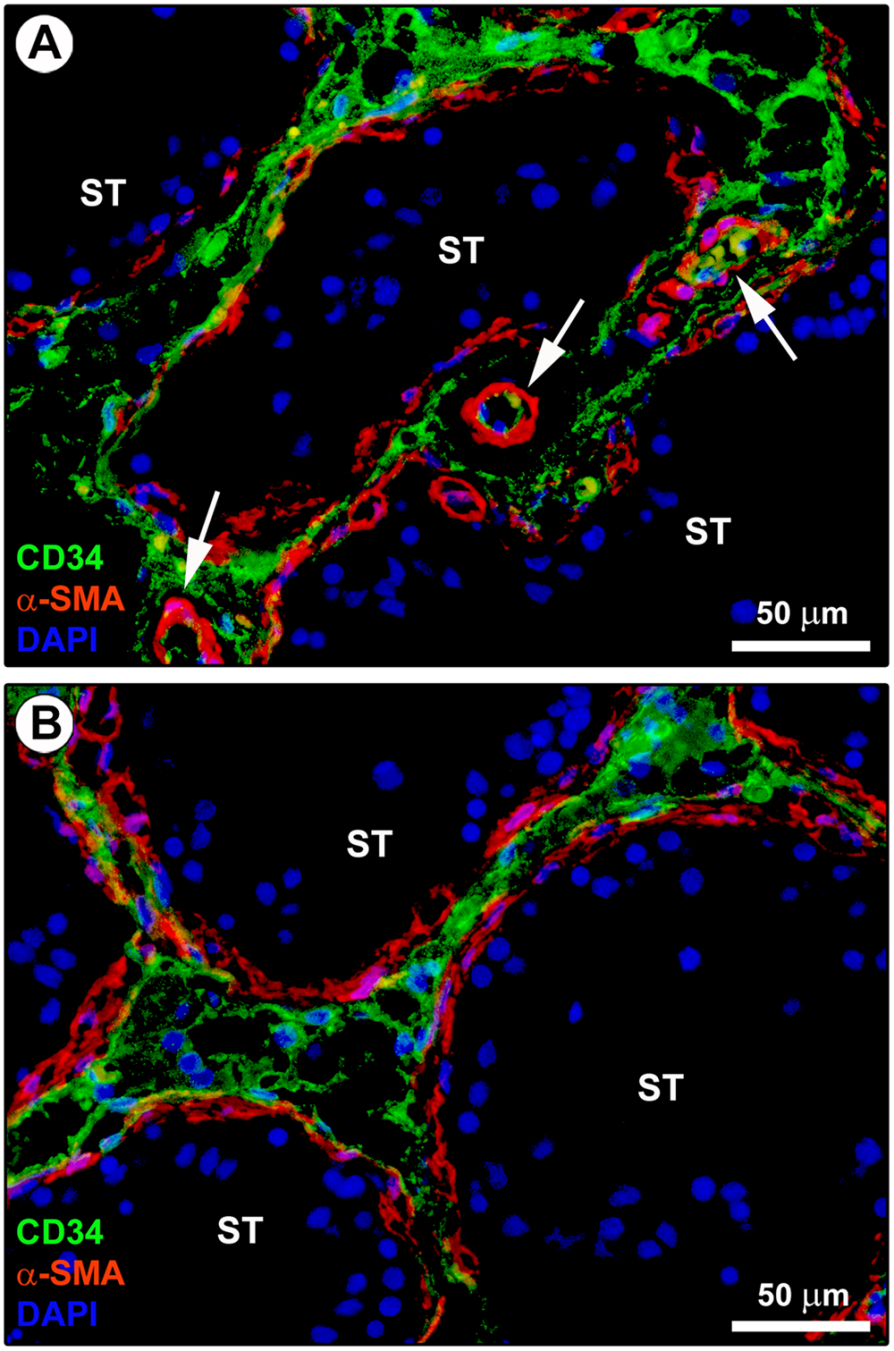
Double immunostaining of human testis sections. (**A,B**) Representative photomicrographs of immunofluorescence labeling for CD34 (green) and α-smooth muscle actin (α-SMA; red). Nuclei are counterstained in blue with 4′,6-diamidino-2-phenylindole (DAPI). CD34-negative/α-SMA-positive myoid cells/myofibroblasts are located in the inner layer of the connective tissue surrounding the seminiferous tubules (ST). Instead, CD34-positive/α-SMA-negative interstitial cells are located in the outer layer of the peritubular tissue. These two cell types form distinct but closely adjacent networks in the peritubular connective tissue; sporadic yellow staining is visible due to apposition of their cellular prolongations. The CD34-positive/α-SMA-negative interstitial cells form a continuous network extending from the outer peritubular tissue to the intertubular stroma. CD34-negative/α-SMA-positive myoid cells/myofibroblasts are absent from the intertubular stroma. α-SMA immunoreactivity is also detected in mural cells (pericytes and smooth muscle cells) of blood vessels (arrows). Scale bar: 50 µm (**A,B**).

**Figure 4:**
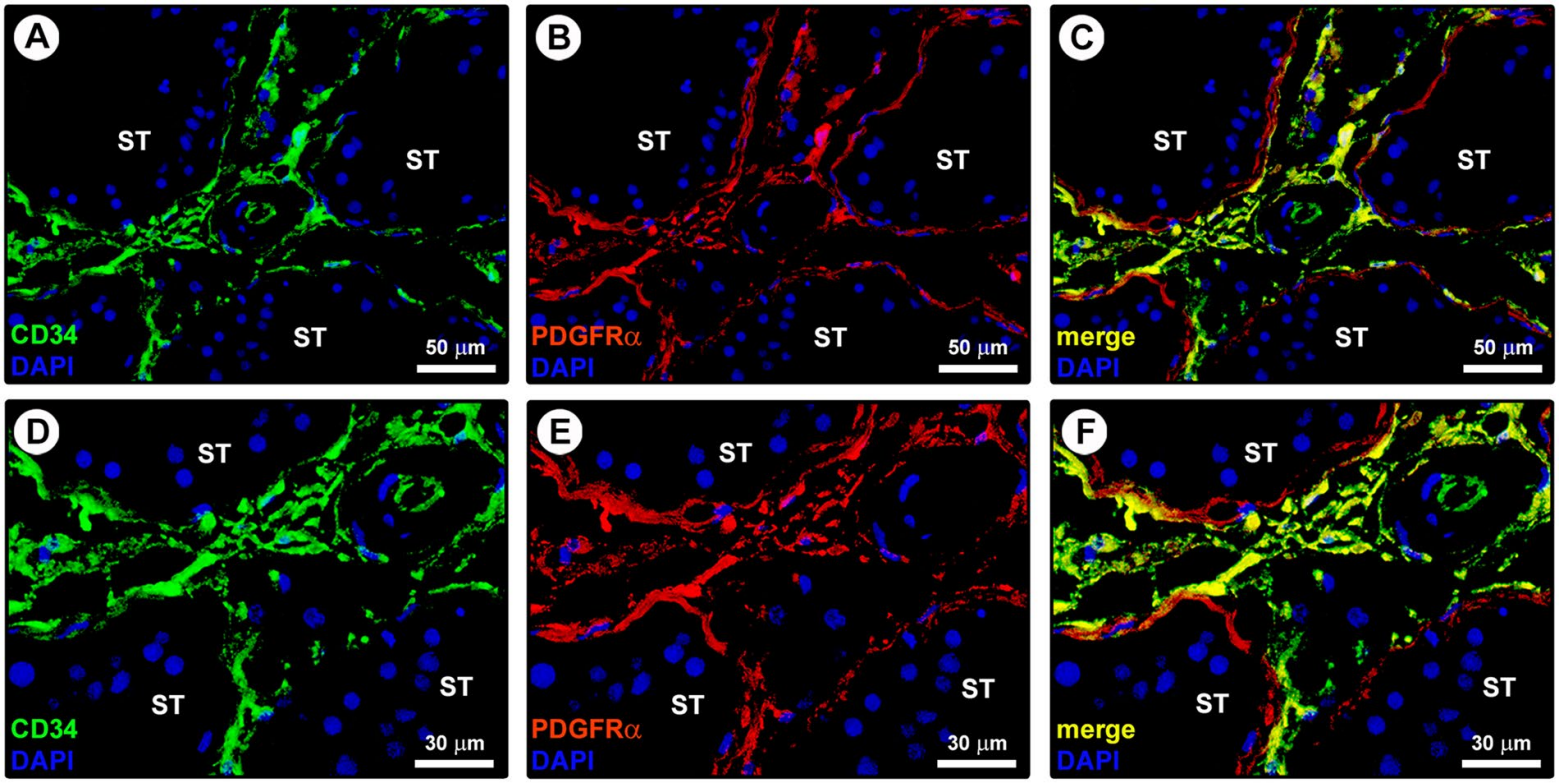
Double immunostaining of human testis sections. (**A–F**) Representative photomicrographs of immunofluorescence staining for CD34 (green) and platelet-derived growth factor receptor α (PDGFRα; red) with 4′,6-diamidino-2-phenylindole (DAPI; blue) counterstain for nuclei. Single green and red images are shown in (**A,D**) and (**B,E**), respectively, while merge images are shown in (**C,F**). All CD34-positive interstitial cells located in the outer layer of the connective tissue around the seminiferous tubules (ST) and in the intertubular stroma coexpress PDGFRα (yellow staining). PDGFRα immunoreactivity is also found in the CD34-negative myoid cells/myofibroblasts located in the inner layer of the peritubular connective tissue. Scale bar: 50 µm (**A-C**), 30 µm (**D–F**).

In line with substantial literature^6,7,16,20,51^, the presence and distribution of TCs in the normal adult human testis were firstly explored by CD34 immunohistochemistry and immunofluorescence. As displayed in Fig. 1A–C depicting representative hematoxylin and eosin-stained tissue slides, these analyses were conducted on paraffin-embedded testicular sections which were carefully selected based on the optimal preservation of the peritubular and intertubular connective tissue testified by the presence of typical clusters of Leydig cells in close vicinity to blood capillaries and embedded in an extracellular matrix. Immunoperoxidase-based immunohistochemistry highlighted that CD34-positive cells were arranged in a complex and continuous network distributed throughout the peritubular and intertubular stromal spaces (Fig. 1D,E). These CD34-positive interstitial cells exhibited an elongated/spindle morphology with long and thin varicose cytoplasmic processes surrounding the seminiferous tubules and forming a reticular network around the Leydig cells and blood microvessels in the intertubular stroma (Fig. 1F–H). Since the CD34 antigen is also expressed by vascular structures that could be misdiagnosed as spindle-shaped interstitial cells especially when appearing as profiles depending on the tissue cutting plane^16^, we further carried out CD34/CD31 double immunofluorescence. As shown in Fig. 2A, the peritubular and intertubular CD34-positive interstitial cells lacked CD31 immunoreactivity, thus making them clearly distinguishable from CD34-positive/CD31-positive vascular endothelial cells. In addition, these CD34-positive stromal cells were immunophenotypically negative for the c-kit/CD117 antigen, whose expression was only detectable in neighboring oval/round-shaped cells, presumably Leydig cells and/or mast cells (Fig. 2B). Keeping into account that spindle-shaped myoid cells/myofibroblasts are well known to be present in the peritubular connective tissue^1,3^, double immunostaining for CD34 and α-smooth muscle actin (α-SMA) was also performed on human testicular specimens. This analysis clearly revealed that two distinct but adjacent cellular entities populated the peritubular interstitium, namely the CD34-negative/α-SMA-positive myoid cells/myofibroblasts located in the inner layer and the CD34-positive/α-SMA-negative cells located in the outer layer of the peritubular stromal compartment (Fig. 3A,B). In particular, the CD34-positive/α-SMA-negative cells formed a continuous network extending from the outer peritubular tissue to the intertubular stroma, where instead the CD34-negative/α-SMA-positive myoid cells/myofibroblasts were absent (Fig. 3A,B). We then characterized more in depth the immunophenotype of these testicular stromal cells by CD34/platelet-derived growth factor receptor α (PDGFRα) double immunofluorescence staining, a cell surface antigenic combination currently considered among the most reliable for the identification of TCs by light microscopy^6,7,16,17,37,46,52–54^. Such double immunolabeling showed coexpression of PDGFRα in all CD34-positive interstitial cells distributed in the outer layer of the peritubular tissue and in the intertubular stroma (Fig. 4A–F). PDGFRα positivity was also detected in the CD34-negative myoid cells/myofibroblasts bordering the basal side of the seminiferous tubules (Fig. 4A–F).

**Figure 5:**
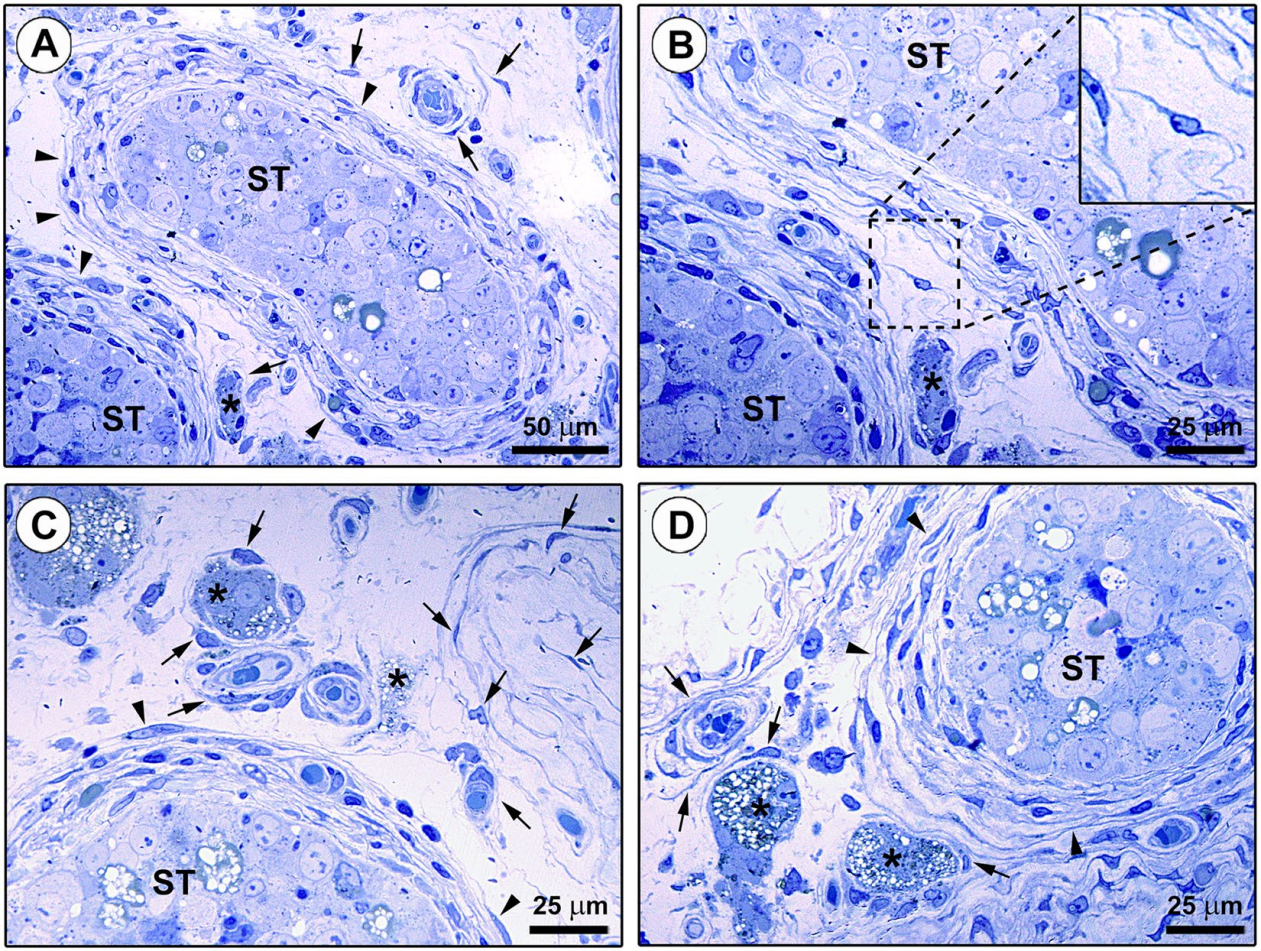
Light microscopy photomicrographs of toluidine blue-stained human testis semithin sections. (**A–D**) Numerous spindle-shaped interstitial cells with very long and thin moniliform cytoplasmic processes are observed in the connective tissue surrounding the seminiferous tubules (ST) (arrowheads). These cells are also broadly distributed in the intertubular stromal space (arrows), where they can be found either in neutral position or around microvessels and steroidogenic Leydig cells (asterisks). The boxed area in (**B**) is shown at higher magnification in the inset; note the bipolar morphology of a spindle-shaped interstitial cell with a cell body containing a large nucleus and very small amount of cytoplasm from which abruptly originate two long and thin moniliform prolongations which are rather convoluted. Scale bar: 50 µm (**A**), 25 µm (**B–D**).

**Figure 6:**
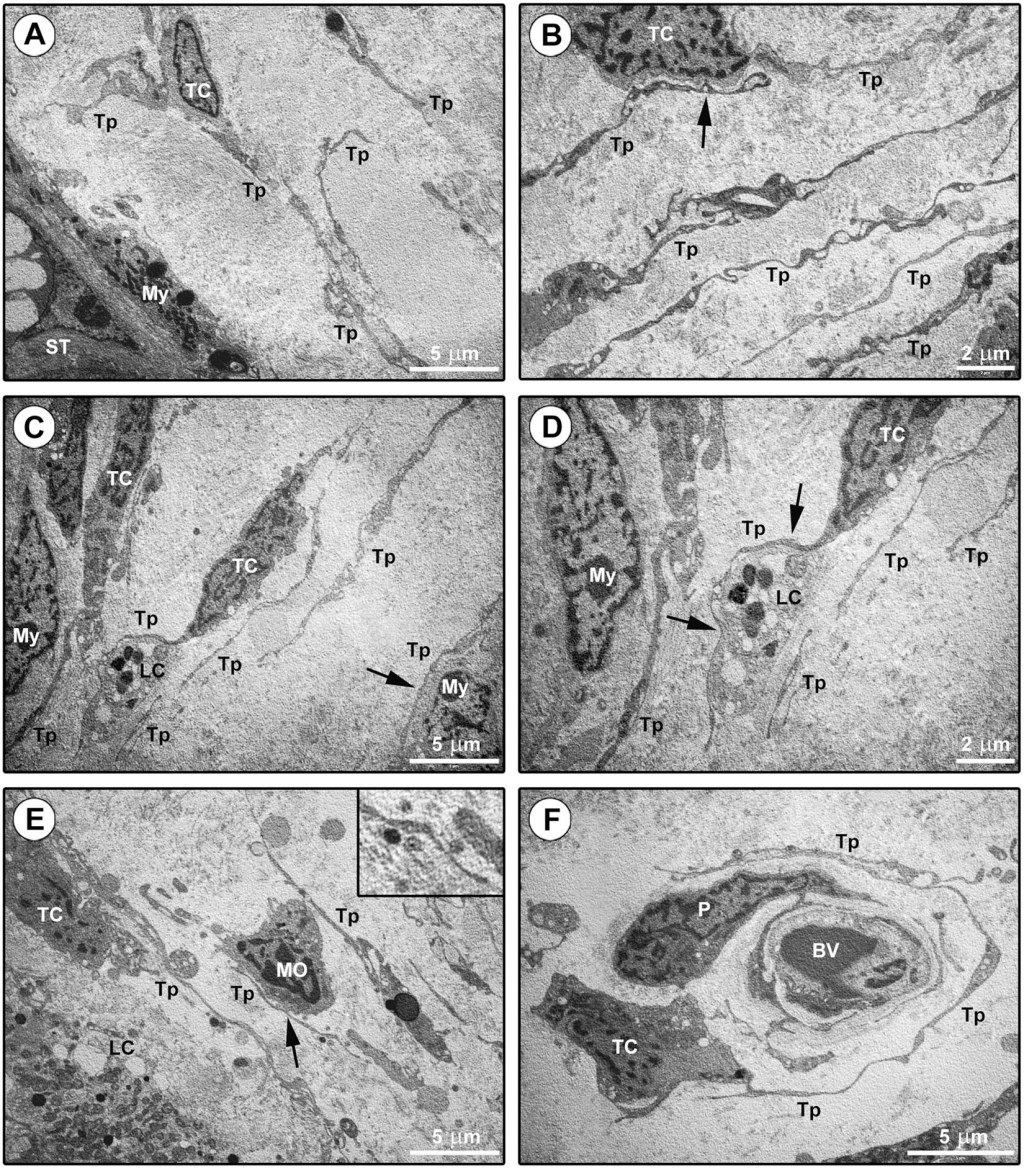
Ultrastructural investigation of the human testis stromal compartment. (**A–F**) Representative transmission electron microscopy photomicrographs of testicular ultrathin sections stained with UranyLess and bismuth subnitrate solutions. Telocytes (TC) are ultrastructurally characterized by (i) a spindle-shaped, oval or piriform cell body mostly occupied by a relatively large euchromatic nucleus surrounded by a scarce cytoplasm, and (ii) the presence of telopodes (Tp), long cytoplasmic processes with a narrow emergence from the cell body and a moniliform silhouette characterized by the alternation of thin segments (podomers) and expanded parts (podoms). (**A**) Telocytes with long telopodes are present in the outer layer of the peritubular connective tissue, while myoid cells/myofibroblasts (My) are located in the inner layer in close contact with the seminiferous tubule (ST) basement membrane. (**B**) Telopodes are often convoluted and form a reticular network in the intertubular stroma. Note an intercellular contact (arrow in **B**) between the cell body of a telocyte and the telopode of another telocyte. (**C–E**) The telopodes of telocytes establish close contacts with peritubular myoid cells/myofibroblasts (arrow in **C**), intertubular steroidogenic Leydig cells (LC) (arrows in **D**) and blood-derived mononuclear cells (MO) (arrow in **E**). Numerous extracellular vesicles are present nearby telopodes (**E**, shown at higher magnification in the inset). (**F**) The long and convoluted telopode of a telocyte intimately surround the abluminal side of the basement membrane of a blood vessel (BV). Note a pericyte (P) embedded in the vessel basement membrane. Scale bar: 5 µm (**A,C,E,F**), 2 µm (**B,D**).

Although the aforedescribed immunohistochemical and immunofluorescence findings were rather evocative of the existence of testicular TCs, we next carried out toluidine blue staining of human testis semithin sections followed by transmission electron microscopy analysis of ultrathin sections to definitively confirm the diagnosis of TCs according to the ultrastructural definition by Cretoiu and Popescu^6^ (Figs 5A–D and 6A–F). Interstitial cells with very long and thin moniliform cytoplasmic processes forming reticular networks in the same tissue locations identified by CD34/PDGFRα immunostaining were observed in toluidine blue-stained testicular semithin sections (Fig. 5A–D). Ultrastructurally, testicular TCs exhibited a spindle-shaped, oval or piriform cellular body containing a large euchromatic nucleus surrounded by a small amount of cytoplasm (Fig. 6A–F). The ultrastructural hallmark of TCs located either in the peritubular or in the intertubular testis stromal space was the presence of telopodes, namely very long, slender and often convoluted cytoplasmic prolongations with a narrow emergence from the cell body and a moniliform silhouette due to the alternation of podomers and podoms (Fig. 6A–F). Intercellular contacts between neighboring TCs and telopodes were frequently observed throughout the testicular stromal compartment (Fig. 6B). Moreover, by their telopodes TCs often established close contacts with peritubular myoid cells/myofibroblasts, intertubular Leydig cells and blood-derived mononuclear cells (presumably macrophages), and intimately surrounded the basement membrane of blood microvessels (Fig. 6C–F). Numerous extracellular vesicles were usually detected nearby telopodes (Fig. 6E, inset).

## Discussion

To the best of our knowledge, the integrated immunohistochemical, immunofluorescence and transmission electron microscopy approach employed in the present study allows us to clearly demonstrate for the first time that stromal cells with distinctive morphological and immunophenotypical features of TCs are broadly distributed within the human testis interstitium. Indeed, based on current recommendations for the identification of TCs^6^, we found that interstitial cells exhibiting typical telopodes (*i.e*. very long processes abruptly emerging from the cellular body and featuring the alternation of slenders podomers and small dilated bead-like podoms) and dual immunoreactivity for CD34 and PDGFRα form a continuous reticular network extending from the peritubular area to the intertubular stromal space of normal testes. Specifically, TCs appear to be located in the outer layer of the peritubular connective tissue, surrounding the inner layer of myoid cells/myofibroblasts. Moreover, the intertubular TC network is in close proximity or even directly connected to blood vessels and Leydig cells. Interestingly, an analogous distribution of TCs has been reported in the testes of the soft-shelled turtle^50^, which is in keeping with the suggested remarkable evolutionary conservation of these peculiar stromal cells across different species in the animal kingdom^55^.

As far as human beings are concerned, cells referred to as CD34-positive stromal fibroblastic/fibrocytic cells have previously been noticed in the testicular stroma^56^. However, although there is mounting evidence that CD34-positive stromal cells may indeed correspond to TCs in a variety of organs^51^, it is essential to keep in mind that (i) the CD34 antigen is not specific to TCs, and (ii) at present only transmission electron microscopy permits the definitive identification of TCs^6^. Based on this assumption, we have taken advantage of both dual immunofluorescence staining and ultrastructural analyses to conclusively verify whether the CD34-positive testicular stromal cells could represent TCs. Indeed, we herein demonstrated the presence of TCs in the human testicular stromal space with the aid of that can presently be considered their most specific immunohistochemical marker combination, namely CD34/PDGFRα double immunofluorescence^6,7,16,17,37,46,52–54^. Furthermore, the combination of CD34 and CD31 immunostaining helped us to undoubtedly discriminate between TCs (CD34-positive/CD31-negative) and the endothelium (CD34-positive/CD31-positive) of blood vessels, and also emphasized the frequent perivascular location of TCs in the intertubular stroma. On the other hand, spindle-shaped α-SMA-expressing myoid cells/myofibroblasts have been previously identified in the connective tissue nearby the seminiferous tubules^3,56^, where we detected an extensive network of CD34/PDGFRα double-positive TCs. Together with recent findings suggesting the possible existence of a subset of α-SMA-positive myoid TCs in some human organs such as the kidney and the urinary bladder^57,58^, the aforementioned evidence prompted us to further carry out CD34/α-SMA double immunofluorescence on human testis samples. Of note, these analyses clearly revealed that the CD34-negative/α-SMA-positive myoid cells/myofibroblasts were selectively located in the inner layer of the peritubular stroma, while the CD34-positive/α-SMA-negative TCs occupied the contiguous outer layer. Collectively, our immunohistochemical data indicated that all human testicular TCs, either in the peritubular or in the intertubular stroma, display the same immunophenotype, that is they are CD34/PDGFRα double-positive and α-SMA-negative. On this basis, we can categorically exclude the existence of a myoid TC subset in the human testis. We also observed that the peritubular CD34-negative/α-SMA-positive myoid cells/myofibroblasts coexpress PDGFRα, which further strengthen the evidence that single immunohistochemistry is not enough and can lead to misleading results when studying TCs by light microscopy. Since the expression of the stemness-related marker c-kit/CD117 has been reported in TCs from some tissues and organs, such as in the heart, skeletal muscles and female reproductive system^6,7,12,14,16,59^, we also double immunolabeled our human testicular sections for CD34/c-kit. Hence, we found that human testicular TCs are c-kit/CD117-negative, as previously documented in other human organs including the gastrointestinal tract and the skin^36,52^. Indeed, we could observe c-kit/CD117 immunoreactivity only in oval/round-shaped stromal cells, presumably Leydig cells and/or mast cells according to literature data^60,61^. At variance with a number of studies^7^, we considered useless to perform vimentin staining, as vimentin can be found almost in every connective tissue cell including fibroblasts, endothelial cells, myoblasts and tissue macrophages among others^62^. Noteworthy, following these in depth immunohistochemical investigations, transmission electron microscopy allowed us to detect cells satisfying the previously established ultrastructural criteria for the identification of TCs^6,20^ in the same locations of the testis interstitium disclosed by the CD34/PDGFRα double labeling. In addition, our electron microscopic observations highlighted that the peritubular and intertubular networks formed by typical telopodes establish intimate relationships with neighboring cells, such as myoid cells/myofibroblasts, endocrine Leydig cells, macrophages and blood capillaries.

Though we are aware of the descriptive/morphological design of the present study, the peculiar spatial TC distribution and their multiple intercellular connections detected in the whole testis stromal space, together with the current knowledge on the presumptive TC roles reported in a variety of organs^6,7,15,16,20,22,26,28,29,41^, allow us to make some interesting speculations on the possible functions of human testicular TCs. For instance, the continuous network of telopodes extending from the peritubular to the intertubular stroma might make a substantial contribution to the morphogenesis and maintenance of the normal three-dimensional architecture of testes. Interestingly, besides their contractile activity, it has been suggested that the peritubular myoid cells/myofibroblasts provide structural support to the seminiferous tubules and are components of the blood-testis barrier^50,63^. Owing to the close spatial association of the telopode network with both the peritubular myoid cells/myofibroblasts and blood vessels, it can be hypothesized that TCs are additional players in the abovementioned functions and likely they participate in the transfer of molecular elements from the interstitial bloodstream to the germinal compartment of seminiferous tubules, thus being potentially involved in the regulation of spermatogenesis. The present study also revealed the presence of numerous TCs that were located in the close vicinity of or even directly connected to Leydig cells by their telopodes, which may suggest that TCs are indirectly involved in the regulation of androgen hormone secretion and release. In this context, it is worth mentioning that the testicular TCs could correspond to the poorly characterized fibroblast-like cells randomly distributed in the intertubular space that have previously been referred to as ‘compartmentalizing cells’ (or ‘Co-cells’) by some authors^1,5^. Besides the similar tissue distribution, the fact that such ‘Co-cells’ were reported to express antigens characteristic for glial cells^1,64^ and that TCs have been hypothesized to be microglial-like cells in a recent study^55^ might, indeed, further support this assumption. According to several studies on TCs in different organs^6,7,16,21–28,33^, the testicular TCs might be engaged in justacrine and/or paracrine signaling and deeply influence the behavior of neighboring cells either by direct junctions or indirectly by extracellular vesicle/exosome release, as here suggested by the numerous extracellular vesicles observed nearby telopodes in the testicular stroma. Taken into account that TCs have also been proposed to behave as ‘hormonal sensors’ in the human female reproductive organs (*i.e*. uterus and fallopian tube) because they express progesterone and estrogen receptors^42,44,46,65^, we cannot exclude an analogue function in the testes. Finally, other presumptive functions of testicular TCs could include a participation in local tissue immune surveillance, guidance of putative stem/progenitor cells and/or representing themselves a pool of tissue resident mesenchymal progenitors^30–32,66^.

## Conclusions

In summary, our study provides the first comprehensive evidence that TCs are part of the microscopic anatomical structure of the human testis. Since these cells appear to be ‘strategically’ positioned forming networks that interplay with multiple cell types within the peritubular and intertubular stromal space, we consider that their possible implications in the physiological and pathological processes of the testes should not be further overlooked. Unveiling the roles exerted by testicular TCs, such as through the investigation of diseased tissues, has also the great potential to shed light on their possible therapeutic utility in the setting of different pathological conditions of human testes.

## Methods

### Human testis specimens

Paraffin- and epoxy resin-embedded normal human testis samples from 12 men aged 18–30 years were selected from the archives of the Section of Anatomy and Histology, Department of Experimental and Clinical Medicine, University of Florence. Archival orchidectomy specimens without evident histopathological lesions were obtained at autopsy (n = 10) or after surgical resection with informed consent (n = 2) as described elsewhere^67^. Immediately after sampling, testicular specimens were divided into small pieces and processed for light and transmission electron microscopy. The study was carried out in accordance with the Declaration of Helsinki and approved by the Hospital Committee for Investigation in Humans (Careggi University Hospital, Florence, Italy).

### Histochemistry and immunohistochemistry

Paraffin-embedded human testis sections (5 µm thick) were deparaffinized and subjected to either routine hematoxylin and eosin staining or immunoperoxidase-based immunohistochemistry for the CD34 antigen using the ready-to-use UltraVision™ Large Volume Detection System Anti-Polyvalent, HRP kit (catalog no. TP-125-HL; Lab Vision, Fremont, CA, USA) according to previously published protocols^17,19,37,39,54^. Briefly, after antigen retrieval in sodium citrate buffer (10 mM, pH 6.0) and blockade of endogenous peroxidases and non-specific antibody binding sites, tissue slides were incubated overnight at 4 °C with a mouse monoclonal anti-human CD34 antibody (1:50 dilution; clone QBEnd-10, catalog no. M7165; Dako, Glostrup, Denmark) or isotype- and concentration-matched irrelevant mouse IgG (Sigma-Aldrich, St. Louis, MO, USA) as negative controls. The day after, tissue sections were incubated sequentially with biotinylated secondary antibodies and streptavidin peroxidase solution (both from Lab Vision) followed by immunoreactivity development using 3-amino-9-ethylcarbazole (catalog no. TA-125-SA; Lab Vision) as chromogen and nuclear counterstaining with hematoxylin. Immunostained tissue sections were examined with a Leica DM4000 B microscope equipped with a Leica DFC310 FX 1.4-megapixel digital color camera and the Leica software application suite LAS V3.8 (Leica Microsystems, Mannheim, Germany).

### Immunofluorescence

Double immunofluorescence staining of paraffin-embedded human testis sections (5 µm thick) combining anti-CD34 with anti-CD31/platelet-endothelial cell adhesion molecule-1 (PECAM-1), anti-c-kit/CD117, anti-α-SMA or anti-PDGFRα antibodies was carried out as described in previous studies^17,36,37,39,54^. After deparaffinization, antigen unmasking in sodium citrate buffer (10 mM, pH 6.0), quenching of autofluorescence and blockade of non-specific antibody binding sites by incubation of testicular tissue sections for 1 hour at room temperature with 1% bovine serum albumin in phosphate-buffered saline, the following primary anti-human antibodies were applied overnight at 4 °C: mouse monoclonal anti-CD34 (1:50 dilution; catalog no. M7165; Dako), rabbit polyclonal anti-CD31/PECAM-1 (1:50 dilution; catalog no. ab28364; Abcam, Cambridge, UK), rabbit polyclonal anti-c-kit/CD117 (1:200 dilution; catalog no. A4502; Dako), rabbit polyclonal anti-α-SMA (1:100 dilution; catalog no. ab5694; Abcam) and goat polyclonal anti-PDGFRα (1:100 dilution; catalog no. AF-307-NA; R&D Systems, Minneapolis, MN, USA). Negative controls were performed using irrelevant isotype- and concentration-matched mouse, rabbit and goat IgG (Sigma-Aldrich). Alexa Fluor-488-conjugated donkey anti-mouse IgG, Rhodamine Red-X-conjugated goat anti-rabbit IgG or Alexa Fluor-568-conjugated donkey anti-goat IgG (all 1:200 dilution; Invitrogen, San Diego, CA, USA) were used as secondary antibodies and nuclei were counterstained with 4′,6-diamidino-2-phenylindole (DAPI). After mounting with an antifade aqueous mounting medium, testis sections were observed under a Leica DM4000 B microscope and fluorescence images were captured with a Leica DFC310 FX 1.4-megapixel digital color camera (Leica Microsystems).

### Transmission electron microscopy

Transmission electron microscopy was carried out as detailed in previously published protocols^17,37^. Epon 812 resin-embedded human testicular specimens were cut with a RMC MT-X ultramicrotome (EMME3, Milan, Italy) and semithin sections (2 μm thick) were stained with a toluidine blue solution in 0.1 M borate buffer and examined under a light microscope. Ultrathin sections (~70 nm thick) of the selected areas were subsequently obtained using a diamond knife and stained with ready-to-use UranyLess solution (Electron Microscopy Sciences, Foster City, CA, USA) followed by an alkaline bismuth subnitrate solution. The stained ultrathin sections were observed and photographed by a high resolution digital camera connected to a JEOL JEM-1010 electron microscope (Jeol, Tokyo, Japan)^17,37^.

## Data Availability

All relevant data are within the paper.
